# The mediating role of cognitive flexibility in home–school co-education and psychological well-being among Chinese vocational college students

**DOI:** 10.3389/fpsyg.2024.1422845

**Published:** 2024-11-08

**Authors:** Chao Liu, Hanjuan Chen, Wenping Xu, Shuling Dai, Shan Lin

**Affiliations:** ^1^School of Medical Technology, Jiangsu College of Nursing, Huai’an, China; ^2^School of Continuing Education, Jiangsu College of Nursing, Huai’an, China; ^3^Department of Medical Technology, Nantong Health College of Jiangsu Province, Nantong, China; ^4^Department of Agricultural Biology and Ecological Technology, Shanghai Vocational College of Agriculture and Forestry, Shanghai, China; ^5^Department of Youth Education and Leadership, Myongji University, Seoul, Republic of Korea

**Keywords:** home-school co-education, cognitive flexibility, depressive symptom, vocational college students, psychological well-being

## Abstract

**Objective:**

This study examines the mediating role of cognitive flexibility in the relationship between home-school co-education (H-SCE) and depressive symptom among vocational college students in urban and rural areas of China.

**Methods:**

Utilizing the Theory of Overlapping Spheres of Influence, this research explores how H-SCE influences depressive symptom, considering cognitive flexibility as a key mediating factor. The study utilized established scales, including the Inventory of Parent and Peer Attachment (IPPA) for parent-child relationships, the Student-Teacher Relationship Scale (STRS), the Cognitive Flexibility Inventory (CFI), and the Center for Epidemiologic Studies Depression Scale (CES-D10).

**Results:**

A total of 1090 valid questionnaires were analyzed, revealing a significant negative correlation between H-SCE and depressive symptom. Cognitive flexibility was found to partially mediate this relationship, suggesting that enhanced cognitive adaptability, fostered through effective H-SCE, can reduce depressive symptom by helping students better cope with stressors.

**Conclusion:**

These findings underscore the importance of fostering strong collaboration between families and schools to enhance cognitive resilience and mitigate psychological challenges faced by vocational students, providing a foundation for targeted interventions to promote mental well-being.

## Introduction

In recent years, the issue of psychological well-being has gained increasing importance globally, particularly in the context of rapid social changes, academic pressures, and the rise of digital technology (Twenge et al., 2021). In China, studies suggest that a significant proportion of students encounter psychological well-being challenges, with reports indicating that nearly 24% of Chinese students experience varying degrees of psychological distress, and a concerning prevalence of depressive symptom is observed (Gao et al., 2020; Gao et al., 2020; Wang et al., 2020). Among these, depression has emerged as a predominant psychological well-being issue confronting students, reflecting a similar trend observed worldwide (Mahmud et al., 2023; Moitra et al., 2022). Specifically, research highlights that vocational college students in China are facing a more severe burden of depressive symptom than their peers in general education, signaling an urgent need for targeted intervention (Wu et al., 2020). Depression, characterized by persistent feelings of sadness, hopelessness, and a lack of interest in previously enjoyable activities (Joyce-Beaulieu and Zaboski, 2021), can severely impair vocational college students’ academic performance and overall well-being (Wagner et al., 2022). It is not just an individual issue, but a societal concern that can result in significant emotional and social repercussions. Recognizing the multifaceted nature of depression is essential, as its roots may lie in various domains, including academic stress, social isolation, and family dynamics. Given the pressing nature of this issue, comprehensive research into the factors that contribute to depressive emotions is imperative for shaping effective psychological education programs. Understanding these influences can foster preventative measures, ultimately aiming to reduce the occurrence of psychological well-being problems among vocational college students and promote a healthier learning environment.

Additionally, the dual economic structure of urban and rural areas in China has long been a topic of scholarly interest, highlighting the stark contrasts between these two environments (Gao, 2021). Urban vocational college students often benefit from greater resources, access to quality education, and social support systems that can enhance their overall psychological well-being (Lee, 2020; Mishra, 2020). In contrast, rural vocational college students frequently face challenges such as limited educational opportunities and fewer mental health resources, leading to disparities in their psychological experiences (Mongelli et al., 2020; Saw and Agger, 2021). As a result, the behaviors and attitudes of vocational college students from urban and rural backgrounds have become vital subjects for investigation in sociology and psychology. Psychological research indicates that urban and rural vocational college students exhibit different social characteristics, which can significantly influence their psychological well-being (Belanche et al., 2021; Liu et al., 2020). Some studies suggest that urban students tend to display higher levels of self-confidence and social assertiveness compared to their rural counterparts, attributed to their advantageous socioeconomic conditions (Vleioras and Robinson, 2022). However, other research contradicts this notion, revealing no significant differences in self-esteem between these groups (Rusnac and Rosciupchin, 2023). Despite the pronounced urban–rural divide in China’s socioeconomic landscape, there remains a notable gap in understanding the specific disparities in depression and psychological well-being issues faced by urban and rural vocational college students. This lack of focused research underscores the necessity for in-depth studies assessing the prevalence and impact of depressive symptom across these two groups. Exploring these differences not only contributes to the academic discourse but also aids in developing tailored psychological well-being interventions that can address the unique challenges faced by vocational college students in varying sociocultural contexts.

### Home-school co-education and psychological well-being

Home-school co-education (H-SCE) is an educational concept that involves jointly fostering students through family education and school education. It establishes a new cooperative relationship among various education stakeholders, including families, schools, and communities, aiming to influence and improve family education practices and family values, promote harmonious coexistence within the community, and achieve coordinated development among families, schools, and communities. This approach seeks to facilitate the collective growth of parents, children, and teachers, allowing them to enjoy a fulfilling and happy educational experience. For the sake of subsequent research and analysis, we simplify H-SCE to focus on parent–child relationships and student-teacher relationships.

The Theory of Overlapping Spheres of Influence (Michael et al., 2007), initially developed by Epstein, emphasizes the interconnectedness of various social systems and their collective impact on an individual’s development. This theory posits that a person’s development is not only influenced by their immediate environment but also by the interactions and relationships among multiple spheres, including family, school, and community. In the context of psychological well-being, and particularly regarding depression, this theory provides a framework to understand how the dynamics of H-SCE can affect mental health outcomes (Lu and Yu, 2024). Depression, a significant mental health issue among individuals, particularly vocational college students, can be examined through the lens of overlapping influences from the home and school environments (Clark et al., 2022; Liu et al., 2022). The interactions within these spheres can either mitigate or exacerbate depressive symptom. For instance, supportive family involvement in education can foster a sense of safety and belonging, potentially reducing the risk of depression (Villatte et al., 2021). Conversely, a lack of communication between home and school settings might lead to feelings of isolation and increased vulnerability to depressive emotions (Schwartz-Mette et al., 2020). Research has consistently demonstrated a correlation between H-SCE practices and the prevalence of depressive symptom among vocational college students (Berryhill and Smith, 2021). Studies indicate that effective collaboration between parents and educators can create a more nurturing environment that promotes emotional resilience (Twum-Antwi et al., 2020). Such findings highlight the importance of understanding the multifaceted influences on mental health and the necessity of fostering strong partnerships between families and schools to enhance vocational college students’ psychological well-being.

H-SCE has emerged as a crucial factor affecting vocational college students’ psychological health, particularly regarding depression, which manifests differently among urban and rural vocational college students. Existing research highlights both direct and indirect influences of H-SCE on depressive symptom, acknowledging the individual differences that exist due to socioeconomic and cultural backgrounds (Lu et al., 2021). For instance, studies have shown that urban students, benefitting from more resources and parental involvement, often experience lower levels of depression compared to their rural counterparts, who may face challenges such as lack of access to mental health support and educational resources (Tan et al., 2020). While the literature has elucidated the relationship between H-SCE and depression, it tends to overlook the role of cognitive flexibility. Cognitive flexibility can significantly mediate the relationship between H-SCE and depression, as variations in cognitive adaptability may lead to differing emotional responses under similar environmental conditions. To address this gap, our research aims to investigate how H-SCE influences depression, considering the mediating role of cognitive flexibility. By exploring this interplay, we seek to uncover the mechanisms through which H-SCE impacts depressive symptom among vocational students from urban and rural areas.

### Cognitive flexibility as a mediator

Cognitive flexibility refers to the mental ability to adapt one’s thinking and behavior in response to new, unexpected, or changing situations. This essential cognitive skill allows individuals to consider multiple perspectives, make decisions based on varying contexts, and adjust their problem-solving strategies accordingly. Empirical research has demonstrated a robust association between cognitive flexibility and mental health, particularly in relation to depression (Huang et al., 2021). Studies indicate that higher levels of cognitive flexibility are linked to decreased depressive symptom, as individuals with this adaptive capacity often exhibit better emotional regulation and resilience in the face of stressors (Rademacher et al., 2023). For instance, research has found that individuals with greater cognitive flexibility are more capable of viewing challenging situations from different angles, which can help them cope more effectively with adversity (Huang et al., 2024). Such adaptability enables them to reframe negative thoughts and reduce rumination, a key factor contributing to depression. By employing cognitive flexibility, these individuals can break free from maladaptive thought patterns, leading to improved mood and emotional health. In light of these findings, enhancing cognitive flexibility through H-SCE frameworks presents a promising approach to mitigating depressive symptom among vocational college students. By fostering effective communication and supportive relationships between families and educational institutions, we can nurture cognitive flexibility in vocational college students. This, in turn, may significantly lower the prevalence of depression among vocational students, equipping them with the necessary tools to navigate both academic challenges and life stressors more successfully.

Cognitive flexibility has been positively correlated with effective teaching and learning processes within the framework of H-SCE. When parents and educators collaborate, students are more likely to develop cognitive flexibility, which enhances their ability to navigate complex situations and promotes better academic and emotional outcomes (Hutchison et al., 2020). Despite the limited research specifically examining the relationship between cognitive flexibility and H-SCE, several studies have suggested a positive connection. For instance, a study by O’Connor and McCartney found that students who experienced consistent communication and support from both home and school environments displayed higher levels of cognitive adaptability (O'Connor and McCartney, 2006). Similarly, research by Rose et al. indicated that H-SCE practices positively influenced students’ adaptive thinking, promoting resilience in stressful situations (Rose et al., 2017). These findings suggest that a nurturing and collaborative educational environment can foster cognitive flexibility among students. However, the investigation into cognitive flexibility’s role in this dynamic remains scarce. To fill this gap, we hypothesize that cognitive flexibility serves as a mediating factor between H-SCE and depression among urban and rural vocational college students (H1). This research aims to explore how enhanced cognitive flexibility derived from effective H-SCE can potentially mitigate depressive symptom, ultimately contributing to better mental health outcomes for vocational college students from urban and rural areas navigating diverse educational contexts.

### The present study

**Figure 1:**
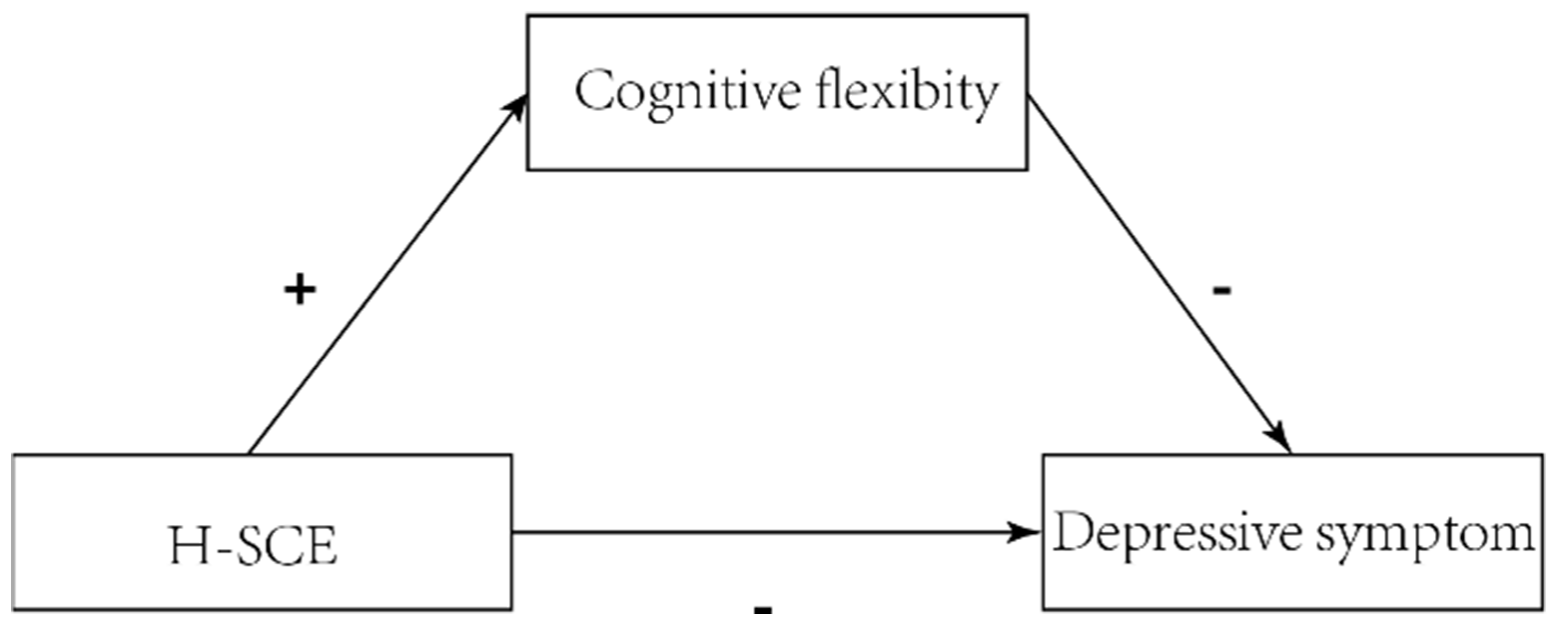
The proposed mediation model (H-SCE: home-school co-education).

Based on the Theory of Overlapping Spheres of Influence, this study delves into the internal mechanisms between H-SCE and depression among urban and rural vocational college students, with a particular focus on the role of cognitive flexibility (see Figure 1). The results are expected to provide theoretical guidance for the prevention of depression in vocational college students.

## Method

### Participants and procedure

The study employed random sampling principles, distributing the questionnaire online using the “Questionnaire Star” platform across five regions of China: eastern, southern, western, northern, and central. This method was designed to ensure a consistent sample size from each region and facilitate centralized collection of the responses. Ethics committee approval was obtained from the college, in line with the declaration of Helsinki (Ashcroft, 2008), and all vocational college students were informed about the study’s purpose prior to the survey. Participants were assured that their personal information and responses would be kept confidential and used exclusively for research purposes. The questionnaire consisted of two primary sections: the first focused on demographic characteristics, while the second covered measurement questions related to the main variables in the study’s model. To ensure reliability and validity, the measurement questions were based on established scales from both domestic and international sources. Inclusion criteria included: (1) a completion time of at least 120 s, (2) avoidance of five consecutive identical responses, and (3) unique internet protocol (IP) addresses. A total of 1,112 questionnaires were collected, with 1,090 classified as valid, resulting in a validity rate of 98.02%.

### Measurements

#### Home-school co-education

The Inventory of Parent and Peer Attachment (IPPA) was used to measure parent–child relationships (Armsden and Greenberg, 1987). This self-report scale consists of 25 items (e.g., “My parents respect my emotions and feelings”) and is divided into three dimensions: communication, trust, and alienation; responses were scored using a 5-point Likert scale (1 = very strongly disagree, 5 = very strongly agree). After reverse scoring certain items within each dimension and computing the average scores, scores from all three dimensions were integrated with equal weight. Higher scores indicate a better parent–child relationship. The IPPA has been widely used with good reliability and validity (Chen et al., 2017), the Cronbach’s *α* for this scale was 0.91.

The abbreviated version of the student–teacher relationships scale (STRS) was used to measure this construct (Pianta and Nimetz, 2001). This scale comprises 15 items (e.g., “My relationship with the teacher is close and warm”) and is divided into two dimensions: closeness and conflict; responses were scored using a 5-point Likert scale (1 = very strongly disagree, 5 = very strongly agree). After reverse scoring certain items within each dimension and computing the average scores, scores from both dimensions were integrated with equal weight. Higher scores indicate a better student–teacher relationship. The STRS has been widely used with good reliability and validity (Aboagye et al., 2019), the Cronbach’s *α* for this scale was 0.93.

#### Cognitive flexibility scale

The cognitive flexibility inventory (CFI) was used to measure individuals’ ability to adapt to the environment and their psychological adjustment ability (Dennis and Vander Wal, 2010). This scale comprises 20 items (e.g., “I consider multiple options before making a decision”); responses were scored using a 5-point Likert scale (1 = very strongly disagree, 5 = very strongly agree). After reverse scoring certain items and computing the average scores, higher scores indicate greater cognitive flexibility. The CFI has been widely used with good reliability and validity (Dennis and Vander Wal, 2010), the Cronbach’s *α* for this scale was 0.96.

#### Depressive symptom scale

The shortened version of the Center for Epidemiologic Studies Depression Scale (CES-D10), developed by Andresen, was used to measure depression (Cheng et al., 2006). This scale consists of 10 items; responses were scored using a 4-point Likert scale ranging from 1 = rarely or none of the time (less than 1 day) to 4 = most or all of the time (5–7 days). A total score of 16 or higher indicates depressive symptom, while a score below 16 is considered normal. The CES-D10 is commonly utilized and demonstrates strong reliability and validity, with a Cronbach’s α of 0.94.

### Statistical analyses

In this study, SPSS 26.0 was employed to evaluate the hypothesis. The Pearson correlation method was applied to assess the relationships among various variables. Additionally, the simple mediation model was examined using Model 4 of the PROCESS macro (Hayes, 2013). A deviation-corrected non-parametric percentile bootstrap program was utilized, with 5,000 repetitions to calculate 95% confidence intervals for assessing the statistical significance of indirect effects.

## Results

### Demographic information

Among the 1,090 valid questionnaires, there were 544 male vocational college students (49.9%) and 546 female vocational college students (50.1%). Age distribution revealed that 127 vocational college students (11.7%) were aged 18 or younger, 631 vocational college students (57.9%) fell within the age range of 19 to 21, and 332 vocational college students (30.5%) were over the age of 21. Regarding family structure, 805 vocational college students (73.9%) identified as only child, while 285 vocational college students (26.1%) reported that they had siblings. Additionally, the financial stability of the respondents’ households was assessed, showing that 878 college students (80.6%) came from families with stable incomes, whereas 212 students (19.4%) were from families with unstable financial situations. When considering geographical background, 838 vocational college students (76.9%) were from urban areas, while 252 vocational college students (23.1%) hailed from rural regions (see Table 1).

**Table 1:** Demographic information.

Variables	Item	Number	Percentage (%)
**Gender**			
	Male	544	49.9
	Female	546	50.1
**Age**			
	18 years and under	127	11.7
	19–21 years	631	57.9
	Over 21 years	332	30.5
**Single-child family**			
	Yes	805	73.9
	No	285	26.1
**Family income stability**			
		878	80.6
		212	19.4
**Household registration**			
	Urban vocational college students	838	76.9
	Rural vocational college students	252	23.1

### Common method bias test

To mitigate the impact of a single sample on inter-dimensional correlations and ensure the scientific validity of the research, Harman’s single-factor method was employed to assess common method bias. The findings indicated that, without rotating the variances of all variables, there were seven factors with eigenvalues exceeding 1. The first factor accounted for 24.32% of the variance, which is below the critical threshold of 40%, suggesting that serious common method bias was not present.

### Preliminary analyses

Pearson correlation analysis was conducted among H-SCE, parent–child relationships, student–teacher relationships, cognitive flexibility, and depressive emotions in the current study (see Table 2). H-SCE, a multidimensional construct, encompasses two key elements: parent–child relationships and teacher-student relationships. The results showed that H-SCE was positively correlated with cognitive flexibility, but depression was negatively associated with H-SCE and cognitive flexibility.

**Table 2:** Descriptive statistics and correlation analysis.

	*M*	SD	1	2	3	4	5
1. H-SCE	3.77	0.52					
2. Parent–child relationships	3.68	0.58	0.90**				
3. Student–teacher relationships	3.92	0.69	0.74**	0.37**			
4. Cognitive flexibility	3.77	0.8	0.37**	0.30**	0.38**		
5. Depressive symptom	21.85	7.07	−0.58**	−0.48**	−0.50**	−0.50**	

H-SCE: home-school co-education, *p < 0.05, **p < 0.01, ***p < 0.001.

### Testing for mediation effects

**Figure 2:**
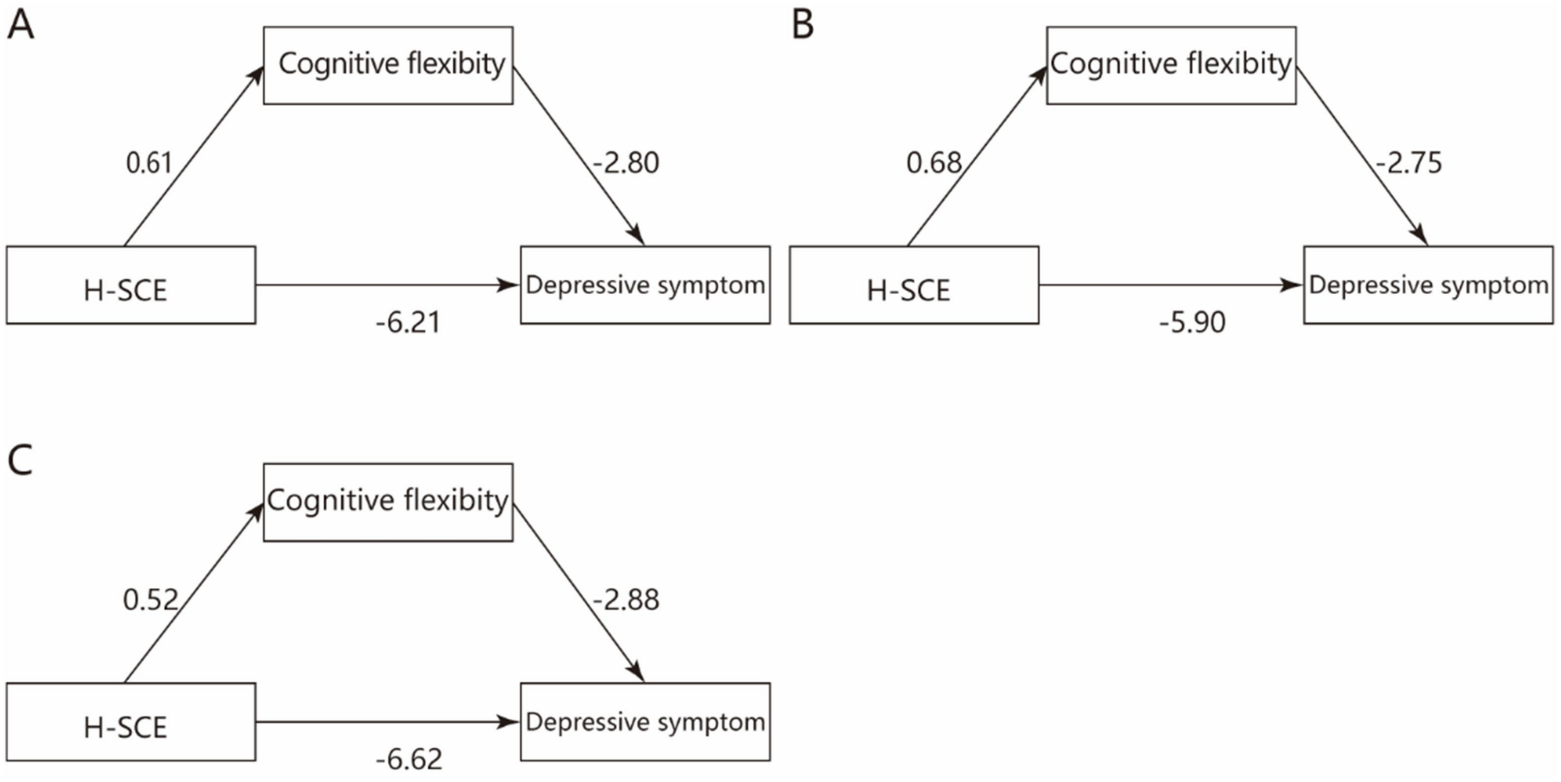
The mediating effect of cognitive flexibility between H-SCE and depression symptom. H-SCE: home-school co-education, (A) the mediating effect of cognitive flexibility between H-SCE and depression symptom among vocational college students, (B) the mediating effect of cognitive flexibility between H-SCE and depression symptom among urban vocational college students, (C) the mediating effect of cognitive flexibility between H-SCE and depression symptom among rural vocational college students.

All variables were standardized prior to analysis and Model 4 in PROCESS was used to examine the mediating effect of cognitive flexibility between H-SCE and depression (see Table 3; Figure 2). The results showed that H-SCE had a significant and negative effect on depression (*β* = −6.21, *p* < 0.001; for urban vocational college students: β = −5.90, *p* < 0.001; for rural vocational college students: β = −6.62, *p* < 0.001), as well as a significant positive effect on cognitive flexibility (β = 0.61, *p* < 0.001; for urban vocational college students: β = 0.68, *p* < 0.001; for rural vocational college students: β = 0.52, *p* < 0.001). Cognitive flexibility had a significantly negative effect on depression (β = −2.80, *p* < 0.001; for urban vocational college students: β = −2.75, *p* < 0.001; for rural vocational college students: β = −2.88, *p* < 0.001). The mediating effect of cognitive flexibility was −1.71 (for urban vocational college students: −1.87; for rural vocational college students: −1.50), accounting for 21.57% (for urban vocational college students: 24.07%; for rural vocational college students: 18.45%) of the total effect. The confidence intervals did not contain 0, indicating that cognitive flexibility partially mediates the relationship between H-SCE and depression.

**Table 3:** Testing the mediating effect of cognitive flexibility between H-SCE and depression symptom.

Predictors	Cognitive flexibility			Depressive symptom		
	*β*	*SE*	95%CI	*β*	*SE*	95%CI
**Total mediation model**						
H-SCE	0.61***	0.06	[0.49, 0.74]	−6.21***	0.52	[−7.23, −5.19]
Cognitive flexibility				−2.80***	0.34	[−3.45, −2.14]
*R* ^2^	0.16			0.42		
*F*	89.84***			172.98***		
**Mediation model for urban vocational college students**						
H-SCE	0.68***	0.09	[0.51, 0.85]	−5.90***	0.67	[−7.21, −4.59]
Cognitive flexibility				−2.75***	0.41	[−3.56, −1.95]
*R* ^2^	0.17			0.42		
*F*	62.55***			105.11***		
**Mediation model for rural vocational college students**						
H-SCE	0.52***	0.10	[0.33, 0.71]	−6.62***	0.83	[−8.26, −4.98]
Cognitive flexibility				−2.88***	0.58	[−4.03, −1.73]
*R* ^2^	0.13			0.42		
*F*	28.42***			67.05***		

H-SCE: home-school co-education, *p < 0.05, **p < 0.01, ***p < 0.001.

## Discussion

In the present study, we examined the role of cognitive flexibility as a mediator in the relationship between H-SCE and depression symptom among vocational college students, focusing on the influence of this dynamic in both urban and rural contexts. The sample consisted of vocational college students, with careful control of demographic variables such as age, gender, whether they were only child, and the stability of their family’s economic background. Utilizing path analysis, we aimed to elucidate the specific mechanisms through which H-SCE impacts vocational college students’ depressive symptom, emphasizing how cognitive flexibility mediates this relationship. Our findings suggest that higher levels of home-school collaboration foster greater cognitive flexibility among vocational college students, which in turn helps mitigate depressive symptom (Shang and Xing, 2021). This indicates that when families and educational institutions work together effectively, vocational college students develop adaptive thinking styles that allow them to better navigate stressors and challenges, ultimately leading to improved mental health outcomes. Particularly for urban and rural vocational college students who may face distinct social and environmental pressures, enhancing cognitive flexibility through supportive H-SCE practices emerges as a vital strategy in reducing the prevalence of depression. Thus, our study underscores the importance of integrating cognitive flexibility training into home-school cooperation initiatives as a means to promote psychological well-being among vocational college students.

### H-SCE and depressive symptom

In examining the relationship between H-SCE and depression symptom among vocational college students from urban and rural backgrounds, our findings reveal a significant negative correlation between these two variables. This association underscores the importance of effective communication and support between families and educational institutions in fostering vocational college students’ psychological well-being (Marcu et al., 2019). Specifically, the data indicates that higher levels of H-SCE correlate with lower levels of depressive symptom, suggesting that collaborative efforts between home and school can effectively buffer against the emotional distress often experienced by vocational college students. This is particularly pertinent in the context of vocational education, where students may face unique stressors related to academic performance, career aspirations, and socio-economic challenges. By enhancing H-SCE, vocational college students may experience a more supportive environment that promotes resilience and adaptive coping mechanisms.

### The mediating role of cognitive flexibility

The present study provides robust evidence supporting the mediating role of cognitive flexibility in the relationship between H-SCE and depression symptom. Specifically, our findings confirm H1, which posits a positive correlation between H-SCE and cognitive flexibility, as well as a negative correlation between cognitive flexibility and depressive symptom (Gabrys et al., 2018). This indicates that vocational college students who experience higher levels of collaboration between their families and educational institutions are more likely to exhibit greater cognitive flexibility. In turn, enhanced cognitive flexibility is associated with lower levels of depression symptom among vocational college students.

These results suggest that H-SCE not only plays a crucial role in supporting vocational college students’ academic and social development but also contributes significantly to their mental health by fostering cognitive adaptability. Cognitive flexibility enables vocational college students to approach challenges from multiple perspectives and develop more adaptive coping strategies, thus buffering against depressive symptom (Dvorakova et al., 2019). The interplay between H-SCE and cognitive flexibility emphasizes the importance of creating supportive environments where families and schools work together to promote vocational college students’ psychological resilience. In essence, our study highlights the potential of targeted interventions that enhance both H-SCE and cognitive flexibility as a means to mitigate depressive symptom among vocational college students. This underscores the necessity for educators and practitioners to prioritize strategies that facilitate effective communication and collaboration between home and school settings as integral components of mental health promotion.

### Limitations, implications, and future directions

The present study faces several limitations that should be acknowledged. Firstly, our sample focused exclusively on vocational college students, which may restrict the generalizability of the findings to broader populations. As a result, the unique characteristics and stressors experienced by this specific group may not accurately reflect the experiences of students in other educational settings. Additionally, given that this is a cross-sectional study, we cannot determine causal relationships between H-SCE, cognitive flexibility, and depression symptom. Future research could benefit from longitudinal studies or experimental designs to further explore the mechanisms that influence mental health over time, thus providing a more comprehensive understanding of these dynamics.

Nevertheless, despite these limitations, this study makes a significant contribution to existing literature and highlights the importance of addressing depression among vocational college students. Theoretically, our research elucidates how H-SCE can effectively reduce depressive symptom in this population, shedding light on the nuances of how and when H-SCE impacts mental health. By evaluating a comprehensive theoretical framework, our findings largely support the hypotheses derived from existing theories, offering empirical evidence for the Theory of Overlapping Spheres of Influence within the Chinese context. This contribution underscores the necessity for educators and policymakers to prioritize H-SCE initiatives as a critical strategy for enhancing the mental well-being of vocational college students, ultimately fostering resilience and promoting healthier academic environments.

## Conclusion

In conclusion, our study explores how H-SCE affects depressive mood while considering the mediating role of cognitive flexibility to fill the gap regarding how cognitive flexibility can significantly mediate the relationship between H-SCE and depression. This study found a negative correlation between H-SCE and depression symptom, a positive correlation between H-SCE and cognitive flexibility, and a negative correlation between cognitive flexibility and depression symptom. Cognitive flexibility also partially mediates the relationship between H-SCE and depression symptom, highlighting its importance in promoting mental health.

## Data Availability

The original contributions presented in the study are included in the article/supplementary material, further inquiries can be directed to the corresponding author.
